# Vitamins C and D and COVID-19 Susceptibility, Severity and Progression: An Evidence Based Systematic Review

**DOI:** 10.3390/medicina58070941

**Published:** 2022-07-15

**Authors:** Filippo Migliorini, Raju Vaishya, Jörg Eschweiler, Francesco Oliva, Frank Hildebrand, Nicola Maffulli

**Affiliations:** 1 Department of Orthopaedic, Trauma and Reconstructive Surgery, RWTH Aachen University Hospital, 52074 Aachen, Germany; joeschweiler@ukaachen.de (J.E.); fhildebrand@ukaachen.de (F.H.); 2 Department of Orthopaedics, Indraprastha Apollo Hospitals Institutes of Orthopaedics, New Delhi 110076, India; raju.vaishya@gmail.com; 3 Department of Medicine, Surgery and Dentistry, University of Salerno, Via S. Allende, 84081 Baronissi, Italy; foliva@unisa.it (F.O.); n.maffulli@qmul.ac.uk (N.M.); 4 School of Pharmacy and Bioengineering, Keele University Faculty of Medicine, Thornburrow Drive, Stoke on Trent ST5 5BG, UK; 5 Barts and the London School of Medicine and Dentistry, Queen Mary University of London, Centre for Sports and Exercise Medicine, Mile End Hospital, 275 Bancroft Road, London E1 4DG, UK

**Keywords:** coronavirus, COVID-19, SARS-CoV-2, susceptibility, vitamin C, vitamin D

## Abstract

*Background and Objectives*: Starting in early December 2019, the novel Coronavirus Disease (COVID-19) from infection with COVID-19 has caused a global pandemic. Many aspects of its pathogenesis and related clinical consequences are still unclear. Early diagnosis and dynamic monitoring of prognostic factors are essential to improve the ability to manage COVID-19 infection. This study aimed to provide an account of the role played by vitamins C and D on the onset, progression and severity of COVID-19. Clinical features and infection-related risk factors are also briefly discussed. *Material and Methods*: In March 2022, the main online databases were accessed. All the articles that investigate the possible role of vitamins C and D on COVID-19 susceptibility, severity and progression were considered. *Results*: The current evidence on vitamin C and D supplementation in patients with COVID-19 infection is inconsistent and controversial. In some studies, vitamins were used as coadjuvant of a formal experimental therapy, while in others as main treatment. Ethnicity and hospital setting (inpatient/outpatient) were also variable. Moreover, there was no consensus between studies in administration protocol: high heterogeneity in dosage, administration, and duration of the treatment were evident. Finally, some studies administered vitamins pre- and/or during COVID infection, in patients with different risk factors and infection severity. *Conclusions*: While waiting to develop a targeted, safe and effective therapy, it is important to investigate individual predisposition and proper disease management. Concluding, available data on the use of nutraceuticals in COVID-19 are inconsistent. However, there is a lack of evidence-based guidelines which recommend vitamin C and D supplementation in patients with COVID-19, and results from high quality randomised controlled trials (RCTs) are inconsistent. Current investigations so far are mostly observational, and include a relatively small sample size which can lead to biased results. Large-scale multicentre studies are therefore needed.

## 1. Introduction

The origins of COVID-19 remain still unclear, with consensus on diagnostic and therapeutic standards [1,2]. In some patients, COVID-19 infection precipitously progresses, and respiratory failure can occur [3]. Therefore, prognostic factors for COVID-19 progression were investigated to provide a basis on which to optimize hospital efforts, further improve patient management and make therapy more effective, hoping to reduce mortality [4,5]. Gender, age, underlying comorbidities (diabetes, hypertension, cardiovascular disease), respiratory rate, temperature, neutrophil and lymphocyte counts, D-dimer, albumin and procalcitonin are all predictors of COVID-19 severity, and therefore can be considered risk factors for admission to intensive care unit (ICU) [6,7,8,9,10,11]. Compared to non-ICU patients, ICU patients had higher plasma levels of IL2, IL7, IL10, GSCF, IP10, MCP1, MIP1A and TNFα [12]. Several risk factors for the progression of COVID-19 pneumonia have been reported, including older age: elderly people are frail, and they are likely to present several comorbidities. These not only increase the risk of pneumonia [13], but also affect prognosis [14]. The assessment of comorbidities is an essential component in determining the prognosis of various diseases, particularly pneumonia [15]. Other risk factors for severity progression are smoking, allergic conditions, asthma, and chronic obstructive pulmonary disease (COPD) [16]. At admission, high fever, dyspnoea, increased C reactive protein, creatinine, and procalcitonin, hypoalbuminemia, leucopoenia, and eosinopenia have been associated with disease progression [17,18,19,20]. Elevated plasmin levels are also common in COVID-19 patients, especially in those with comorbidities [21]. Plasmin or other proteases can increase COVID-19 severity by accelerating the entry, fusion, duplication and release of the virus in respiratory cells. Elevated plasmin could be an independent factor for risk stratification of patients with COVID-19 [22,23]. Therefore, patients presenting with these characteristics need to be monitored more carefully to avoid complications and poor prognosis. COVID-19 pneumonia is characterized by acute onset and rapid progression [24]. Lung damage caused by such infections can progress to acute respiratory distress syndrome (ARDS) [25]. Recently, vitamin C and D supplementation in patients with COVID-19 has gained interest. However, their role in the onset, progression and severity of the disease in COVID-19 is still unclear. Therefore, this systematic review investigated whether vitamin C and D supplementation may play a role in reducing the susceptibility, severity and progression of COVID-19 infection. The hypothesis of the present investigation is that the administration of vitamins C and D in COVID-19 patients might positively impact disease susceptibility, severity and progression.

### 1.1. The Role of Vitamin D

Vitamin D plays a major role in calcium regulation and bone health [22]. Vitamin D also acts on immune cells, and is generally able to reduce inflammation [26,27]. Vitamin D is a potent epigenetic regulator, affecting more than 2500 genes [28] and playing a role in a variety of diseases, including cancer [29,30], diabetes mellitus [31], acute respiratory tract infections [32] and autoimmune diseases such as multiple sclerosis [33]. According to the World Health Organisation (WHO), the world has recorded more than 140 million cases and more than 3 million deaths related to COVID-19 since its outbreak [34]. Some aspects suggest a potential association between vitamin D and COVID-19 [35]. The first is seasonality, as the infection started in winter in the Northern Hemisphere and mortality rates were lower in the summer, especially in Europe, only to pick up again in September in various European countries: mortality appears to be inversely related to solar ultraviolet-B (UVB) doses and vitamin D production [36,37]. The second is ethnic group: African and Hispanic Americans experienced higher rates of COVID-19 infection and death rates than European Americans [38,39], possibly from darker skin pigmentation and lower concentrations of 25(OH)D [40]. Further aspects are systemic inflammation and immune dysregulation mediated by COVID-19, which are associated with vitamin D on the immune system [41,42]. Vitamin D modulates the innate immune response and has antiviral activity and is therefore recommended to prevent acute respiratory infections [43,44]. Hypovitaminosis D increases the risk of infectious diseases, supported by the hypothesis that seasonal variations in acute respiratory infections could be related to seasonal variations in vitamin D levels [45,46,47,48].

### 1.2. The Role of Vitamin C

The investigation into the role of vitamin C in the prevention and treatment of pneumonia and sepsis has been ongoing for many decades. This research has laid a solid foundation to transpose the findings to patients with severe COVID-19 [49]. Patients with pneumonia, sepsis and multiple organ failure have low vitamin C status and high oxidative stress [50,51,52]. These critically ill patients have a higher requirement for vitamin C, requiring gram doses to normalize their blood levels [53,54], 20–30 times greater than what is required for the general population. Intravenous administration of vitamin C to patients with pneumonia to normalize plasma levels is an intervention which, as suggested by some studies, is able to reduce the severity (days spent in ICU) and duration (hospital stay) of the disease [55,56,57,58,59,60]. Despite these findings, critically ill patients with sepsis continue to receive milligram amounts of vitamin C, which is insufficient to replenish their vitamin C status [61]. Vitamin C has pleiotropic physiological functions, many of which are relevant to COVID-19. These include antioxidant, anti-inflammatory, antithrombotic and immunomodulatory functions [62]. Many of the functions of vitamin C seem relevant for the cytokine storm, sepsis and ARDS related to COVID-19 [63,64,65,66,67,68].

## 2. Materials and Methods

### 2.1. Search Strategy

This systematic review was conducted according to the Preferred Reporting Items for Systematic Reviews and Meta-Analyses: the 2020 PRISMA statement [69]. The literature search was conducted following this protocol:

- Patients: COVID-19 infection;
- Comparison: vitamins C and D;
- Outcomes: susceptibility, severity and progression of COVID-19.

### 2.2. Eligibility Criteria

All the comparative clinical studies which investigated the role of vitamin C and D supplementation or deficiency in COVID-19 susceptibility, severity and progression were considered in the present systematic review. The studies were eligible irrespective of the clinical severity of the infection and/or of the presence and severity of patients’ comorbidities and/or protocol of vitamins administration. Studies level I to IV of evidence, according to the Oxford Centre of Evidence-Based Medicine [70], were considered. Grey literature, abstracts and posters, comments, expert opinion and editorials were not considered in the quantitative analysis. Given the authors’ language capabilities, articles in English, French, German, Italian and Spanish were considered. Disagreements were solved by a third author (N.M.).

### 2.3. Literature Search 

In May 2022, the following databases were accessed: Pubmed, Embase, Scopus and Google Scholar. The following keywords were used for the search using the Boolean operator AND/OR: (coronavirus OR COVID-19 OR SARS-CoV-2 OR SARS-CoV-1 OR pandemic) AND (vitamins OR vitamin C OR vitamin D) AND (administration OR supplementation OR exogenous OR factors OR predictors OR susceptibility OR prognosis OR diagnosis OR role OR therapy) AND (onset OR severity OR progression OR outcome OR sepsis OR pneumonia OR lung OR cytokines OR pneumonia OR pathogenesis OR response OR risk OR death OR mortality OR morbidity OR inflammatory OR nutraceutical OR immune system). The database search was performed without time constrains. If title and abstract matched the topic, the full text was accessed. The bibliographies of the full-text articles were also screened for inclusion.

### 2.4. Methodology Quality Assessment

The Newcastle–Ottawa Scale (NOS) was used to assess the quality of non-randomized studies. The NOS uses a ‘star’ rating system to judge the quality of studies, and is based on selection, comparability and assessment of outcome. The maximum number of stars a study may receive is nine, attributed as follows: zero to four stars for selection, zero to two stars for comparability and zero to three stars for the outcome. A final score ≥6 stars indicated a good quality. The Jadad composite scale was used to assess the methodological quality of the clinical trials based on randomization, blinding and withdrawals. The scale ranged from zero to five points. A final score ≥3 stars indicate low risk of bias.

## 3. Results

### 3.1. Search Results

**Figure 1:**
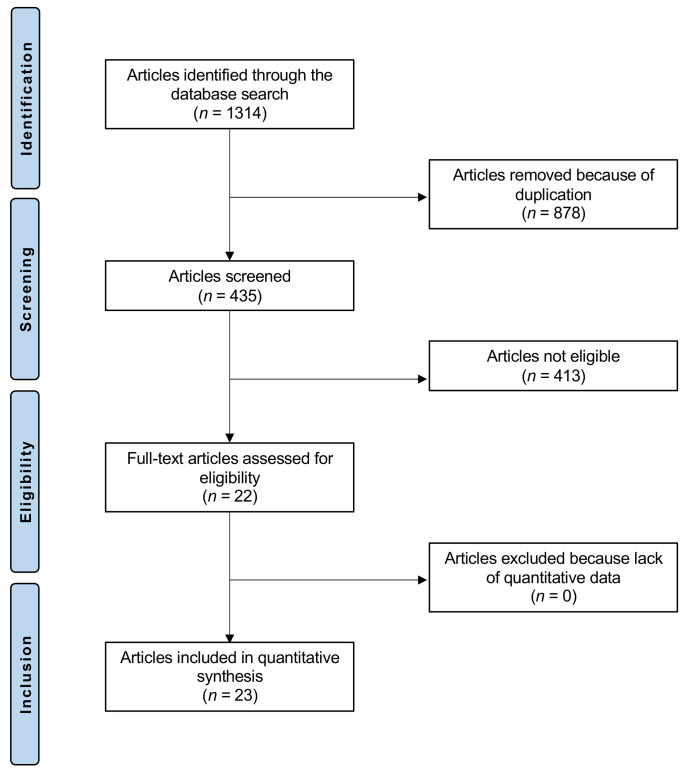
Flow chart of the literature search.

The literature search resulted in 1314 articles. Of them, 878 were excluded, as they were duplicates. A further 413 were excluded with reason: not clinical investigation (*n* = 174), not focusing on Vitamin D or C (*n* = 201), not focusing on COVID (*n* = 30), language limitation (*n* = 8). This left 23 studies for the present investigation (Figure 1).

### 3.2. Syntheses of Results

Thirteen studies compared Vit D supplementation in patients with COVID (Table 1). Overall, 3443 patients were included: 830 in the treatment group and 2581 in the control group. Men made up 50% (1722 of 3443). The mean age of the patients was 59.6 ± 13.9 years old. Several clinical investigations found that in patients hospitalized with COVID-19, administration of vitamin D was associated with lower in-hospital mortality compared to a control group who did not receive supplementation or received low-dose supplementation [71]. These results were also confirmed in the elderly, along with a reduced severity progression compared to a control group who did not receive the vitamin supplementation [72,73,74,75]. Other studies found that vitamin D supplementation improved oxygenation [76] and reduced the need of ICU in inpatient regimes [77]. On the other hand, other clinical investigations found no difference in the outcome in patients who received Vitamin D supplementation in terms of severity of disease in need of mechanical ventilation, length of hospital stay and in-hospital mortality compared to those who did not [78,79,80,81].

Nine studies compared Vit C supplementation in patients with COVID (Table 2). Overall, 1488 patients were included: 605 in the treatment group and 883 in the control group. Men made up 52% (774 of 1488). The mean age of the patients was 59.8 ± 7.4 years old. High-dose vitamin C may reduce the mortality [85] and the rate of thrombosis [86], and improve oxygenation [85,87], in patients with COVID-2019. Other studies did not find benefits for vitamin C in addition to the main treatment regimen in inpatient and outpatient regimes [86,88,89,90,91,92,93], with no impact on mortality, need for mechanical ventilation [89], nor vasopressor requirements or Sequential Organ Failure Assessment (SOFA) scores [90].

### 3.3. Methodological Quality Assessment

The NOS resulted ≥6 stars in all studies, and the Jadad composite scale ≥3 in most studies. These results attested to the present study’s good quality concerning the methodological assessment.

The methodological quality assessment of the non-randomized investigations is shown in Table 3 and that of the randomised controlled trials in Table 4.

## 4. Discussion

According to the main findings of the present study, our hypothesis that the administration of vitamins C and D in COVID-19 patients impacts positively on disease susceptibility, severity and progression was not supported by the current evidence. The current evidence on vitamin C and D supplementation in patients with COVID-19 infection is heterogeneous. Although most studies focused on the same endpoints, such as infection progression, hospitalisation length, oxygenation, need of ICU and mechanical ventilation, and mortality, the results of these studies are controversial, and no consistent recommendation can be inferred. The current clinical investigations evidenced high variability in eligibility criteria, patient comorbidities and age, and associated therapies. In some studies, vitamins were used as coadjuvant of a formal experimental therapy, while in others as main treatment. Ethnicity and hospital setting (inpatient/outpatient) were also variable. Moreover, there was no consensus between studies in administration protocol: high heterogeneity in dosage, administration and duration of the treatment were evident. Finally, some studies administered vitamins pre- and/or during COVID infection, in patients with different risk factors and infection severity.

The improved metabolism of vitamins in diseases and inflammatory processes may justify vitamin C and D supplementation during sepsis or pneumonia. However, the possibility of decreasing the incidence of viral diseases in a population using dietary supplements of vitamin C and D is not supported by the present published peer reviewed literature. This is in accordance with previous evidence, which concluded that vitamin administration at high doses does not contribute to reducing the severity and progression of COVID-19 [49,94,95]. Among other things, the basal state of vitamin C probably influences the response to its administration. As recently highlighted, most patients who were enrolled in the clinical studies suffered from hypovitaminosis, and such hypovitaminosic patients are more likely to respond to its therapeutic supplementation [49,94,96]. We were unable to identify studies which investigated the efficacy of vitamin C and D supplementation in the prevention of COVID-19 in healthy patients. In this context, the effects of vitamin C and D application are not fully generalisable. While waiting to develop a targeted, safe and effective therapy, it is important to investigate individual predisposition and proper disease management [97]. Available data on the use of nutraceuticals in COVID-19 are inconsistent. Irrespective of COVID-19 infection, vitamins have beneficial properties with practically no side effects. Prevention of malnutrition by providing adequate amounts of macronutrients to maintain energy needs is highly recommended [98]. Integration with micronutrients is equally important to prevent viral infections: low levels of vitamins have been associated with adverse clinical outcomes [99]. Given the good safety profile, low cost, and the potential for rapid increase in their production, the administration of vitamins C and D to patients with infectious diseases may be reasonable. However, there is a lack of evidence-based guidelines which recommend vitamin C and D supplementation in patients with COVID-19, and results from high quality randomised controlled trials (RCTs) are inconsistent. Current investigations so far are mostly observational, and include a relatively small sample size which can lead to biased results. Large-scale multicenter studies are therefore needed. Vitamins participate in a multitude of biochemical pathways which may affect the susceptibility, severity and progression of COVID-19. Higher serum concentrations of 25(OH)D contribute to maintaining intact the epithelial layers, reducing virus replication, modulating pro-inflammatory cytokine and promoting the concentration of free angiotensin-converting enzyme-2 (ACE-2). These effects of vitamin D may explain the positive results obtained in some studies. More research is needed to evaluate the mechanisms by which Vitamin D may reduce the risk of COVID-19. Vitamin C shows relevant mechanisms of action for severe respiratory infections, including antioxidant, anti-inflammatory, antithrombotic and immunomodulatory functions. However, whether low concentrations of either vitamin C or vitamin D were a causative reason or an unrelated collateral effect related to COVID-19 susceptibility, severity and progression remains unclear. Whether exogenous replacement for higher serum levels of vitamins C or D would result in better prevention or better treatment outcomes is debated, and relevant evidence is missing. The results from larger RCTs currently would provide more definitive evidence. Optimization of intervention protocols in future trials, for example, an early and prolonged administration, is justified to ascertain their effectiveness. Concluding, the current available evidence is inconsistent and not exhaustive; therefore, vitamins C and D in COVID-19 patients should be cautiously administered under continuous monitoring.

The hypothesis that vitamin D status may affect the risk of COVID-19 is mainly based on observational studies [100,101,102,103,104,105,106,107,108,109,110,111,112,113,114]. We were unable to identify RCTs which investigated the incidence of COVID-19 in patients underoing vitamin D supplementation. Most studies which have identified inverse correlations between COVID-19 severity and risk of death are retrospective with limied numbers of COVID-19 patients. To date, the United States Observational Study is the largest observational study, reporting data on 191,779 patients with values of vitamin D tested in the previous 12 months [115]. The study reported COVID-19 positivity rates vs vitamin D concentration [115]. The finding that the COVID-19 positivity rate in the United States varied from 6.5% for vitamin D concentrations between 40 and 50 ng/mL, to 11.3% for values of 20 ng/mL, may result from the effect of vitamin D in reducing virus survival and replication by induction of cathelicidin and defensins, as well as by increasing free ACE-2 concentrations, thus preventing COVID-19 from entering cells through the ACE-2 receptor [116]. Regression fit to all data indicates that COVID-19 positivity is 40% lower for 25(OH)D values above 50 ng/mL (Institute of Medicine recommended value [117,118]) compared to values of 20 ng/mL. The higher rates in the northern states were explained by a genetic variation of the original Chinese form of the amino acid D614A of the Spike protein, to the mutated European form D614G [119]. The variation of the Spike amino acid is caused by a nucleotide mutation from A to G in position 23,403 of the Wuhan reference strain. The D614G form has higher transmission capabilities, and was introduced to New York by people returning from Europe. Such genetic alteration possibly explains some of the high COVID-19 positivity rates in these areas. Regarding ethnic differences, social determinants predisposing to COVID-19, such as lower income, reduced education and employment, as well as higher rates of comorbidities might explain why African Americans have higher rates of COVID-19 [120]. These factors may help explain why blacks and Hispanics have COVID-19 positivity rates 7% and 4% higher, respectively, than whites for the same vitamin D (30 ng/mL). However, the positive spread rate of COVID-19 was higher for blacks and Hispanics with 20 ng/mL than for whites with the same value (18%, 16% and 9%, respectively) than for blacks and Hispanics with about 60 ng/mL (11%, 9% and 5%, respectively), suggesting that low vitamin D status plays a role in increasing the rate of COVID-19. A potential limitation of Vitamin D studies is some scientific evidence that acute inflammatory disease lowers the concentration of 25(OH)D [121,122,123,124,125]. Another important factor which might be relevant in COVID-19 infection severity and progression is the interaction of vitamin D and its receptor (vitamin D receptor, VDR), as vitamin D is an important immunomodulator of both innate and adaptive immune responses [126,127]. Some previous investigations concluded that vitamin D supplementation might be effective to modulate COVID-19 infection at the early and at the hyperinflammatory stages [77,102,128]. Despite these positive results, the efficacy of Vitamin D application in patients with COVID-19 still remains controversial, and future studies are required [129]. A phase 3 RCT, the CORONAVIT study is currently ongoing (NCT04579640). In this study the authors determined whether population-level implementation of a test-and-treat approach to correction of sub-optimal vitamin D status influences the risk of acute respiratory infection or COVID-19 disease. In adult sub-optimal vitamin D status, vitamin D implementation has no impact on the risk of acute respiratory infection or COVID-19 disease.

Regarding vitamin C, many observational studies indicated a low state of vitamin C in critically ill patients with COVID-19 [130,131]. Currently, several RCTs are evaluating intravenous administration of vitamin C monotherapy [132,133,134,135]. Given the pharmacokinetics of vitamin C and the increased requirement during pulmonary infections, oral vitamin C might not be as effective as intravenous administration [136], significantly reducing levels of IL-6 by the first week [87]. Coagulopathy and development of microthrombi represent further common complications of COVID-19 [63], which probably are important components of lung problems [137]. Early vitamin C injections prevent microthrombi formation and blockage of capillaries [138]. Indeed, previous studies have also shown that the level of D-dimer in COVID-19 patients decreased if intravenous vitamin C was administered [139]. Another possible complication of COVID-19 is the extracellular neutrophil traps, which may predispose to the coagulopathy related to COVID-19 [140,141]. In sepsis models, administration of vitamin C may decrease the extracellular neutrophil trap formation [142]. Neutrophil-derived oxidative stress is thought to induce tissue damage in COVID-19 [143,144]. Patients with respiratory infection and sepsis demonstrated a considerable elevation of the oxidative stress markers compared to other critical patients [145,146]. In these patients, administration of vitamin C stabilized markers of oxidative stress, improving the survivorship [56,147]. Moreover, the administration of vitamin C in critical patients might have shortened the mechanical ventilation and the length the intensive care stay [148,149]. The above findings may be particularly important in countries with limited capacity of ICUs beds and more generally in contexts with limited resources, such as low-middle-income countries [150]. Most of the top 10 countries with the highest number of cases for COVID-19 have low-medium income [151], a known risk factor for hypovitaminosis C [152].

## 5. Conclusions

According to the main findings of the present study, our hypothesis that the administration of vitamins C and D in COVID-19 patients impacts positively disease susceptibility, severity and progression was not supported by the current evidence. While waiting to develop a targeted, safe and effective therapy, it is important to investigate individual predisposition and proper disease management. Available data on the use of nutraceuticals in COVID-19 are inconsistent. However, there is a lack of evidence-based guidelines which recommend vitamin C and D supplementation in patients with COVID-19, and results from high quality randomised controlled trials (RCTs) are inconsistent. Current investigations so far are mostly observational, and include a relatively small sample size, which can lead to biased results. Large-scale multicentre studies are therefore needed.

## Figures and Tables

**Table 1:** Studies which investigated Vit D implementation in COVID.

Author, Year	Country of Origin	Design	Setting	Follow-Up (Days)	Hospitalisation (Days)	Patients (Treatment/Control)	Mean Age	Male (%)
Acala-Diaz et al., 2021 [71]	Spain	Retrospective	Inpatient	30	NR	79/458	67.3 ± 15.9	21
Annweiler et al., 2020 [72]	France	Prospective	Inpatient	14	NR	45/32	88.4 ± 5.3	51
Annweiler et al., 2020 [73]	France	Prospective	Nursing & residents	36	NR	9/57	87.7 ± 9.0	23
Annweiler et al., 2022 [82]	France	RCT	Inpatient	28	NR	127/127	88 (82 to 92)	42
Arroyo-Diaz et al., 2021 [78]	Spain	Cross-sectional	Inpatient	NR	7.86 ± 8.5	89/1078	64.7 ± 16.3	55
Cangiano et al., 2021 [74]	Italy	Retrospective	Nursing & residents	60	NR	20/78	89.9 ± 6.5	29
Cereda et al., 2021 [83]	Italy	Retrospective	Out & inpatient	NR	NR	38/286	70.3 ± 12.8	49
Castillo et al., 2020 [77]	Spain	RCT	Inpatient	NR	NR	50/26	53.01 ± 10.24	59
Elamir et al., 2020 [76]	USA	RCT	Inpatient	NR	5.5 ± 3.9	25/25	66.5 ± 17.04	50
Guven et al., 2021 [79]	Turkey	Retrospective	Inpatient	NR	9 (6 to 16)	113/62	74 (61 to 82)	60
Hernandez et al., 2020 [80]	Spain	Case control	Inpatient	11.8 ± 6.1	NR	19/197	59.4 ± 16.8	57
Jevalikar et al., 2021 [81]	India	Cross-sectional	Inpatient	NR	NR	128/69	46.7 ± 18.8	68
Lakkireddy et al. 2021 [75]	India	RCT	Inpatient	NR	13 ± 5	44/43	45 ± 13	75
Murai et al., 2021 [84]	Brazil	RCT	Inpatient	7.7	7 (4 to 10)	44/43	56.2 ± 14.4	56

RCT: randomised controlled trial; NR: not reported.

**Table 2:** Studies which investigated Vit C implementation in COVID.

Author, Year	Country of Origin	Design	Setting	Follow-Up (Days)	Hospitalisation (Days)	Patients (Treatment/Control)	Mean Age	Male (%)
Al Sulaiman et al., 2021 [86]	Saudi Arabia	Retrospective	Inpatient	NR	8.5 (5 to 15)	148/148	60.6 ± 15.15	68
Gao et al., 2021 [85]	China	Retrospective	Inpatient	28	NR	46/30	61 (52 to 71)	39
Jamali Moghadam et al., 2021 [88]	Iran	RCT	Inpatient	NR	8.50 (7 to 12)	30/30	59.3 ± 17.1	50
Kumari et al., 2020 [89]	Pakistan	RCT	Inpatient	NR	8.1 ± 1.8	75/75	52.5 ± 11.5	57
Li et al., 2021 [90]	USA	Retrospective	Inpatient	NR	18 ± 13	8/24	64.7 ± 10.9	38
Suna et al., 2021 [91]	Turkey	Retrospective	Inpatient	NR	8.1 ± 4.2	153/170	62.3 ± 14.2	63
Thomas et al., 2021 [92]	USA	RCT	Outpatient	28	NR	48/50	43.8 ± 14.8	35
Zhang et al., 2020 [87]	China	RCT	Inpatient	28	35 ± 17	27/29	66.7 ± 12.7	64
Zheng et al., 2021 [93]	China	Retrospective	Inpatient	29.3	NR	70/327	67 (61 to 74)	52

RCT: randomised controlled trial; NR: not reported.

**Table 3:** Newcastle–Ottawa Scale.

Author, Year	Selection	Comparability	Outcome	Score
Alcala-Diaz et al., 2021 [71]	4	1	2	7
Al Sulaiman et al., 2021 [86]	4	1	2	7
Annweiler et al., 2020 [72]	4	1	2	7
Annweiler et al., 2020 [73]	4	1	1	6
Arroyo-Diaz et al., 2021 [78]	4	0	2	6
Cangiano et al., 2021 [74]	4	1	3	8
Cereda et al., 2021 [83]	4	1	1	6
Gao et al., 2021 [85]	4	1	2	7
Guven et al., 2021 [79]	4	1	2	7
Hernandez et al., 2020 [80]	4	0	2	6
Jevalikar et al., 2021 [81]	4	1	1	6
Li et al., 2021 [90]	4	1	1	6
Suna et al., 2021 [91]	4	1	1	6
Zheng et al., 2021 [93]	4	1	3	8

**Table 4:** Jadad Composite Scale.

Author, Year	Randomisation	Blinding	Withdrawals	Score
Annweiler et al., 2022 [82]	2	0	1	3
Castillo et al., 2020 [77]	2	0	1	3
Elamir et al., 2020 [76]	2	0	1	3
Jamali Moghadam et al., 2021 [88]	2	0	0	2
Kumari et al., 2020 [89]	2	0	0	2
Lakkireddy et al., 2021 [75]	2	0	1	3
Murai et al., 2021 [84]	2	2	0	4
Thomas et al., 2021 [92]	2	0	1	3
Zhang et al., 2020 [87]	2	0	1	3

## Data Availability

Not applicable.
